# The role of secondary features in serial dependence

**DOI:** 10.1167/jov.23.5.21

**Published:** 2023-05-26

**Authors:** Christian Houborg, Árni Kristjánsson, Ömer Dağlar Tanrıkulu, David Pascucci

**Affiliations:** 1 Vision Sciences Laboratory, School of Health Sciences, University of Iceland, Reykjavik, Iceland; 2 Vision Sciences Laboratory, School of Health Sciences, University of Iceland, Reykjavik, Iceland; 3 Vision Sciences Laboratory, School of Health Sciences, University of Iceland, Reykjavik, Iceland; 4 Department of Psychology, University of New Hampshire, Durham, NH, USA; 5 Laboratory of Psychophysics, Ecole Polytechnique Fédérale de Lausanne, Switzerland

**Keywords:** serial dependence, sequential biases, object processing, attention

## Abstract

Recent work indicates that visual features are processed in a serially dependent manner: The decision about a stimulus feature in the present is influenced by the features of stimuli seen in the past, leading to serial dependence. It remains unclear, however, under which conditions serial dependence is influenced by secondary features of the stimulus. Here, we investigate whether the color of a stimulus influences serial dependence in an orientation adjustment task. Observers viewed a sequence of oriented stimuli that randomly changed color (red or green) and reproduced the orientation of the last stimulus in the sequence. In addition, they had to either detect a certain color in the stimulus (Experiment 1) or discriminate the color of the stimulus (Experiment 2). We found that color does not influence serial dependence for orientation, and that observers were biased by previous orientations independently of changes or repetitions in the stimulus color. This occurred even when observers were explicitly asked to discriminate the stimuli based on their color. Together, our two experiments indicate that when the task involves a single elementary feature such as orientation, serial dependence is not modulated by changes in other features of the stimulus.

## Introduction

In our everyday visual environment, we are constantly exposed to a myriad of visual features. A major challenge for the visual system is to represent the history of these features in a meaningful way (i.e., determining which feature corresponds to the same stimulus at different times). Recent work on serial dependence suggests that visual features are temporally combined over an extended region of time and space, in a way that promotes the continuity of feature processing (for a review, see Pascucci et al., 2023). This notion stems from evidence of serial dependence in almost any sort of visual task, where current decisions about a stimulus feature are biased toward the features of stimuli seen before (see Cicchini & Kristjánsson, 2015; Kiyonaga, Scimeca, Bliss, & Whitney, 2017). This systematic bias has often been related to the way perception combines sequential views and promotes the continuity of object representations (Fischer, Czoschke, Peters, Rahm, Kaiser, & Bledowski, 2020; Kiyonaga et al., 2017; Liberman, Zhang, & Whitney, 2016).

However, the extent to which serial dependence can be related to object processing remains unclear. The majority of studies have, indeed, focused on investigating serial dependence on a single dimension of stimuli, when only one feature changes such as orientation. Objects are, by definition, made of combinations of features that co-occur, maintain a constant relationship, and remain stable for a certain period of time (Treisman, 1996; Treisman & Schmidt, 1982).

But, results on the effects of varying multiple features in serial dependence have been quite mixed. For example, Fritsche and de Lange (2019) showed that the feature that causes serial dependence is the one that was attended to on the previous and current trial. When the size of a stimulus was attended on the preceding trial, the previous stimulus orientation had a reduced effect on current judgments about orientation (Fritsche & de Lange, 2019). Similarly, Collins (2021) showed that, when the relevant feature is the stimulus shape, changes in the stimulus orientation can reduce but not prevent serial dependence in size judgments. Additionally, when the task involves the orientation of the stimulus, and a secondary feature such as color or the pattern of the stimulus is completely irrelevant, changes in the secondary feature do not affect serial dependence (Ceylan, Herzog, & Pascucci, 2021; Huffman, Pratt, & Honey, 2017; Kim, Burr, Cicchini, & Alais, 2020). Other work suggests that, when a secondary feature (e.g., color) is relevant to the primary task (e.g., motion discrimination), serial dependence occurs for stimuli that share the secondary feature (Fischer et al., 2020). Moreover, when the task involves more complex stimuli, such as judgments about faces, which are defined by a combination of local features, serial dependence may occur at the object level and, therefore, for combinations of features (Collins, 2021; Liberman, Manassi, & Whitney, 2018).

Taken together, the above studies seem to suggest that the effect of a secondary feature on serial dependence is a function of the relevance of that feature for ongoing processing. When the secondary feature can be completely ignored it has no effect, or it may only modulate but not determine serial dependence. These findings seemingly complicate a clear picture of the relationship between serial dependence and object continuity, because objects maintain the relationship between their defining features (Pascucci et al., 2023). One possibility is that, in experimental settings, the explicit instructions to focus on a single feature encourage observers to ignore other aspects of the display, thus preventing binding of multiple features across perceptual episodes.

We investigated the role of an additional task that requires the processing and discrimination of a secondary feature in serial dependence. By having observers focus on both the primary and secondary features, we evaluated whether serial dependence would be modulated by the secondary feature, in a way consistent with object-based processing. We used a variant of a classic paradigm in which multiple stimuli are sequentially presented within a single trial and only the last stimulus is reported (i.e., a “sequential no-report” task) (Pascucci et al., 2023). This paradigm has previously been used to disentangle two forms of serial dependence: one induced by the stimulus, in the absence of behavioral report, and one induced by the last reported stimulus (Pascucci, Mancuso, Santandrea, Libera, Plomp, & Chelazzi, 2019; Pascucci & Plomp, 2021). This corresponds to measuring the effect of the last stimulus within the trial sequence (within-trial) and the effect of the stimulus reported before (between-trial), terminology that we will use to distinguish the two throughout the manuscript. The within-trial sequence had variable length and consisted of a series of Gabor stimuli with varying orientations and two possible colors, red or green. Observers performed two tasks. In Experiment 1, they had to reproduce the orientation of the last stimulus at the end of the sequence and quickly detect the occurrence of stimuli of the designated target color within the sequence (by pressing a key). The target color always corresponded to the color of the last Gabor. Hence, the color defined the target dimension for both the detection task and the orientation adjustment task. In Experiment 2, observers again reproduced the orientation of the last Gabor but discriminated between the color of each Gabor during the sequence. Hence, color was relevant for the discrimination task but irrelevant for the orientation task. Our main question was whether asking observers to actively use the color feature would modulate serial dependence in a way consistent with object-level processing—that is, only the orientation of stimuli possessing the same color would be subject to serial dependence. If this were the case, serial dependence would only be observed between stimuli of the same color, in both Experiments 1 and 2. Conversely, if serial dependence is totally independent of the secondary task, then the orientation of previous stimuli would induce a bias on current judgments independently of the color tasks and manipulations, in both experiments.

Our results revealed a more nuanced picture. In Experiment 1, serial dependence was modulated by color, but this most likely occurred because color cued the orientation to attend. In Experiment 2, where both colors were attended and not informative about the relevant orientation, serial dependence occurred completely independently of color. Furthermore, in both experiments, we found strong biases toward the orientation reported on the preceding trial, independently of the color and of the fact that many additional stimuli were shown within the current trial. We discuss these results in light of the role of attention, task relevance, and object processing in serial dependence.

## Methods

### Ethics statement

The study was performed in accordance with the requirements of the local ethics committee.

### Apparatus

Stimuli were generated with custom-made scripts in MATLAB R2021a (MathWorks, Natick, MA) and the Psychophysics Toolbox (Brainard, 1997), and presented on a 24-inch monitor (resolution: 1920 × 1080 pixels, refresh rate: 60 Hz; Asus, Taipei, Taiwan), on a Windows-based machine (Microsoft, Redmond, CA). The experiment was performed in a quiet and dimly lit experimental booth, and participants were positioned approximately 57 cm from the computer screen.

### Participants

Forty-one healthy human participants (age range, 18–52 years; nine females; 19 subjects in Experiment 1 and 22 in Experiment 2) from the University of Iceland took part in the study for a monetary reward (1500 ISK). Participants had normal or corrected-to-normal vision and were naïve as to the purpose of the experiments. Written informed consent was collected from all participants beforehand.

### Stimuli and procedures

**Figure 1.**
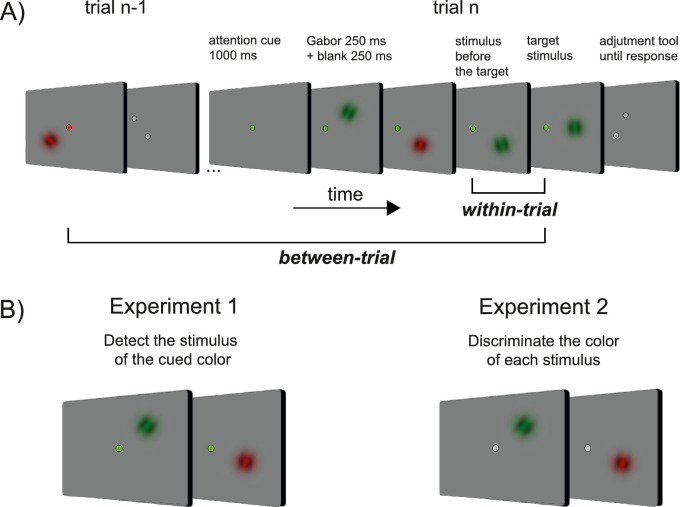
The paradigm and the main experimental manipulations (stimuli are not drawn to scale). **(A)** Each trial contained a sequence of oriented Gabors whose color (red or green) was randomly assigned. Observers were asked to perform a task during the sequence (see B) and to reproduce the orientation of the last stimulus when the sequence ended. The orientation reproduction was made by rotating an imaginary line connecting two dots. **(B)** In Experiment 1, the task during the sequence was to detect and quickly report the occurrence of a stimulus with the same color as the fixation dot. In Experiment 2, the task was to indicate whether the present stimulus was red or green by pressing two corresponding keys. The fixation dot had a neutral color in Experiment 2.

An example of a trial sequence in Experiments 1 and 2 is depicted in Figure 1. The experiments consisted of a series of trials containing a sequence of stimuli. Each trial started with a fixation dot shown for 1000 ms. A sequence of Gabors (peak contrast of 100%, spatial frequency of 0.75 cpd, and a Gaussian envelope of 25°) was then presented. Each Gabor was presented for 250 ms and followed by a 250-ms interstimulus interval. On a single trial, there could be 4, 6, or 8 Gabors, presented at random locations, with their center positioned on the circumference of an imaginary circle (radius of 8° from the fixation dot). The length of the sequence (4, 6, or 8) was randomly determined on each trial. The fixation dot remained on the screen for the entire duration of each trial, and its color varied between Experiments 1 and 2 (Figure 1B, and see below). The Gabors presented in a sequence were red or green, and the color was randomly determined. A response tool appeared at the locus of the fixation point 250 ms after the presentation of the last Gabor. The response tool consisted of two dark-gray dots positioned at the extremities of an imaginary line, and participants had to rotate the line to reproduce the perceived orientation of the last Gabor. The initial orientation of the response tool and the orientation of all of the Gabors were selected randomly, with the exception that the maximum orientation difference between Gabors in a single trial sequence was fixed at ±50°. Between trials, the orientation difference could vary within the whole ±90° angular range.

Before each experiment, participants were provided with written and verbal instructions, and they performed a sequence of practice trials under the supervision of the experimenter. Practice trials were not analyzed further. Both experiments were divided into 10 blocks of 20 trials and lasted approximately 1 hour. Participants were instructed to maintain their gaze at the center of the screen for the entire duration (breaks excluded). Participants were free to take breaks between each block. All stimuli were presented on a gray background (83.33 cd/m^2^).

#### Experiment 1

Experiment 1 had 19 participants (four excluded, see below). In this experiment, the color of the fixation dot was red or green (Figure 1B). During the sequence of Gabors presented on each trial, participants had to press the “X” key as fast as possible whenever a Gabor of the same color as the fixation dot was detected (*target*), while ignoring (i.e., not responding to) Gabors of the irrelevant color (*non-target*). At the end of the sequence, they reproduced the orientation of the last seen Gabor with an adjustment response. The color of the last Gabor, at the end of the sequence, always matched the color of the fixation dot, so the last Gabor was always task relevant. Participants were informed at the beginning of the experiment that the fixation color also indicated the color of the stimuli relevant to the orientation adjustment task.

#### Experiment 2

Experiment 2 had 22 participants (six excluded, see below). In this experiment, the color of the fixation dot was gray and had no relationship with the relevant stimuli in the orientation task; therefore, all stimuli were task relevant. During the sequence, participants had to discriminate the color of each Gabor by pressing a related response key (“X” for red, “C” for green). At the end of the sequence, they reproduced the orientation of the last seen Gabor, which could equiprobably be red or green.

### Data preprocessing

#### Outlier exclusion

Before the main analysis, trials were marked as outliers and removed in the following cases: (1) accuracy in the within-trial detection or discrimination tasks was lower than 50%, or (2) absolute adjustment errors were larger than 30° (Cicchini, Mikellidou, & Burr, 2018) or adjustment times were slower than 10 seconds. Trials at the beginning of each block were also removed, leading to a total proportion of trials removed of less than 9% for Experiment 1 and 12% for Experiment 2. For subject exclusion, we applied the following criteria: (1) more than 25% of their trials were marked as outliers, or (2) a value of circular correlation between the reported and presented orientation was lower than 0.5. Ten subjects were excluded based on these criteria (four in Experiment 1 and six in Experiment 2).

#### Orientation bias removal

Orientation space is not perceptually uniform, and notable biases have been documented (Appelle, 1972; Balikou et al., 2015; van Bergen & Jehee, 2019). This is particularly relevant for the analysis of serial dependence, and previous work has recommended corrections for these biases to avoid unwanted noise (Sheehan & Serences, 2023). To remove such biases, orientation adjustment responses were first demeaned and then residualized from oblique effects and orientation biases. Sinusoidal (Pascucci et al., 2019) or polynomial (Manassi, Liberman, Kosovicheva, Zhang, & Whitney, 2018; Rafiei, Hansmann-Roth, Whitney, Kristjánsson, & Chetverikov, 2021) fits have been used in previous work to capture systematic orientation biases, but there is no standard procedure. Here we used a nonlinear mixed model with fixed and random effects estimated on the whole group of subjects. We started from the assumption of a sinusoidal trend in orientation biases (Balikou et al., 2015; Pascucci et al., 2019; Sheehan & Serences, 2023; van Bergen & Jehee, 2019) and fitted a cumulative sum of sinusoidal functions with a frequency increase of one cycle. The model fitting proceeded with the recursive addition of sinusoids with increasing frequency (from one to six cycles over the 0°–180° orientation range), as both fixed and random effects. The resulting set of coefficients quantified the best-fit amplitude of each sinusoid at the population level (fixed effects) and individual level (random effects). At each step, we computed the circular correlation between the residuals of the model and the orientation variable. Sinusoidal functions with an estimated amplitude lower than 1 and providing only a negligible increase in correlation were then discarded. This resulted in the choice of four sinusoidal functions (one, two, four, and six cycles) to best model the orientation bias. The individual residuals obtained from this model were used for the rest of the analyses.

### Statistical analysis of serial dependence

Following the approach of Barbosa and Compte (2020), we presented the average curves using folded errors, computed by multiplying trial-wise error by the sign of the trial-wise relative difference in orientations (Δ). The average folded errors were then plotted as a function of absolute values of Δ. For the serial dependence analyses, we used a single-trial, nonlinear, mixed-effects model following the same approach as in Pascucci et al. (2019). Briefly, individual and single-trial residualized adjustment errors were fitted to the first derivative of a Gaussian (δoG) function with amplitude (α) and width (*w*) as free parameters. In the *within-trial* analysis, we modeled serial dependence as a function of the inducer color—that is, whether the Gabor stimulus before the last one was of the *target* or *non-target* color. In doing so, the mixed-effects model included two δoG functions multiplied by a dummy variable coding for the condition (Pascucci et al., 2019), for a total of four parameters (two amplitudes and widths per condition). All parameters were included as fixed and random effects. The statistical significance of individual parameters and comparisons between parameters were computed employing *t*-tests and *z*-tests, respectively (Pascucci et al., 2019). A separate model with a similar structure was used in the analysis of *between-trial* serial dependence, with the dummy variable coding whether the color of the previously reported stimulus was the *target* as on the present trial or a *non-target*. In this latter analysis, responses following trials marked as outliers were also excluded. All mixed-effects models were estimated using *nlmefit.m* (with *fminunc* as the optimization function) from the Statistics and Machine Learning Toolbox (MATLAB R2021a). Initial parameter guesses were α = 2° and *w* = 0.05. Note that, even though the plots were made with smoothed and folded average errors for graphical purposes, the model fit and results were performed on single-trial errors; thus, the smoothing factor and folding procedure did not influence the model results.

## Results

### Experiment 1

Fifteen observers were presented with a sequence of four, six, or eight low-contrast Gabors on each trial. The Gabors were either green or red (randomly intermixed) and were presented briefly in the periphery of the visual field (see methods, and Figure 1A). Each trial started with a colored fixation dot that indicated the color of the *target* stimulus in the sequence, and the other color was designated the *non-target* stimulus.

**Figure 2.**
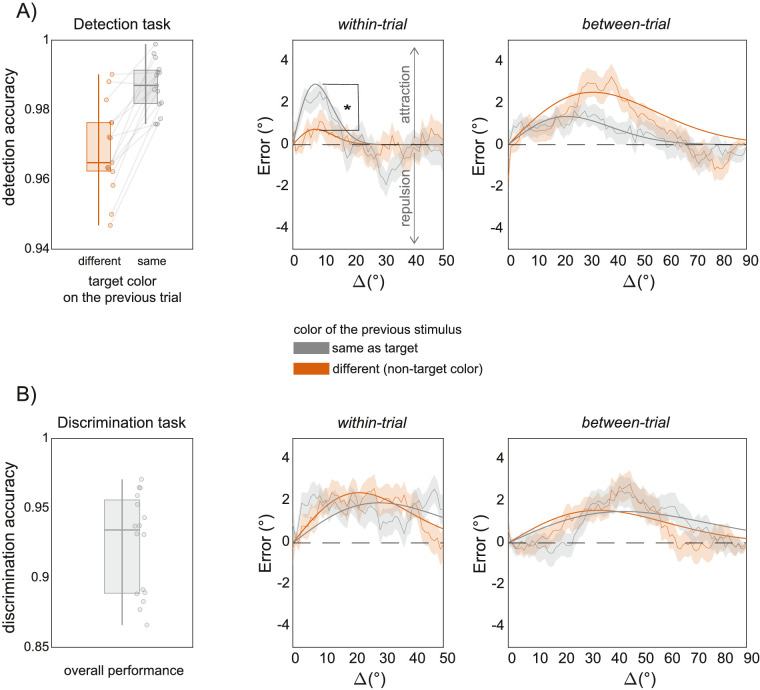
Main results of the two experiments. **(A)** In Experiment 1, observers detected the occurrence of Gabor stimuli with the *target* color indicated by the fixation dot, while ignoring stimuli of the *non-target* color. Performance on the detection task was strongly influenced by the repetition of the same target color—an intertrial-priming effect (left panel). *Within-trial* serial dependence was only evident when the previous stimulus in the trial sequence had the *target* color (i.e., the same color as the last stimulus to be reported), whereas *non-target* stimuli produced no effect (middle panel). *Between-trial* serial dependence occurred independently of the stimulus color (right panel). **(B)** In Experiment 2, observers discriminated the color of each stimulus in the sequence. Discrimination accuracy was well above chance (left panel). In this experiment, both *within-* and *between-trial* serial dependence were significant, independently of the color of the stimulus. Note that, in Experiment 2, *target* and *non-target* refer to whether or not the previous stimulus (within or between the trials) was of the same color as the one to be reported. Curves depict a running average ± 1 SD of adjustment errors over Δ (using a sliding window of 9° for the *within-trial* and 19° for the *between-trial* plots, for graphical purposes). Fitted lines depict the predictions of the best-fitting δoG for each condition.

Overall, participants performed the detection task with an accuracy of 98%, also showing strong inter-trial priming effects (Kristjánsson & Ásgeirsson, 2019): The detection rate increased when the color of the target stimulus was the same on two consecutive trials; for the same versus different color, *t*(14) = −5.98, 95% confidence interval = −0.0248 to −0.0117, *p* < 0.001 (see Figure 2A). This priming effect indicated that the memory of the target color on the previous trial persisted on the present trial.

Participants performed the orientation adjustment task with a standard deviation of errors of 8.36° ± 6.47° and an average adjustment time of 2.12 ± 1.04 seconds. The analysis of serial dependence focused on whether the last reported (*between-trial*) and last seen (*within-trial*) Gabor had the *target* or *non-target* color. In principle, if observers combine orientations depending on the stimulus color, then both within- and between-trial serial dependence should occur for stimuli of matching color, resembling the observed priming effect. Conversely, if the only feature that matters is the relevant orientation, then within-trial serial dependence should be induced by stimuli of the target color, because the target color indicates the relevant orientation, whereas between-trial serial dependence should be independent of color changes (e.g., a stimulus of a different color could be the target on the previous trial).

In the *within-trial* analysis, orientation adjustment responses showed the typical serial dependence pattern only when the previous stimulus had the *target* color (Figure 2A, within-trial, gray curve and line). In this condition, response errors were biased toward the orientation of the last seen Gabor, particularly when the difference in orientation between the previous and present stimulus (Δ) was small, leading to a significant half-amplitude of the δoG curve (α = 2.90°, *p* < 0.001, two-tailed *t*-test of the nonlinear mixed-model parameter α against 0; see Methods). Conversely, previous stimuli with *non-target* colors produced no bias (α = 0.74°, *p* > 0.05). The difference between *target* and *non-target* serial dependence was significant (*target* minus *non-target*, α difference = 2.16°, *p* = 0.013, *z*-test on the parameters’ difference) (see Figure 2A).

In the *between-trial* analysis, however, the orientation reproduced on the last trial induced a strong bias on current responses, independently of whether the color attended on the previous trial was the *target* or *non-target* on the present trial (see Figure 2A). When the target color was the same on two consecutive trials, current responses were significantly biased toward the previously reported orientation (α = 1.35°, *p =* 0.002). The results were similar when the target color on the preceding trial was different from the current one, with a qualitatively larger serial dependence amplitude (α = 2.51°, *p <* 0.001), which did not differ significantly across conditions (*target* minus *non-target*, α difference = −1.15°, *p* = 0.103).

### Experiment 2

In Experiment 1, observers were biased by the orientation of the target color within-trial and by the orientation reported on the preceding trial, independently of the color. This finding may have two explanations. The first is that only relevant and attended stimuli cause orientation serial dependence, no matter the stimulus color. In this case, serial dependence was modulated by the target color during the sequence because the target color indicated the orientation to attend, but it was independent of the color between trials because stimuli of different colors could be targets on different trials. The second is that the effects measured within and between trials reflect different forms of serial dependence, one driven by the last stimulus (within-trial) and the other by the last report (between-trial). In this case, it is still possible that within-trial serial dependence was modulated by the stimulus color. However, because we did not test the bias in reproducing stimuli of the non-target color, the results cannot determine whether stimuli were combined depending on the color or simply because of the relevant orientation.

In Experiment 2, we therefore tested within-trial serial dependence for Gabors of both colors, making them both relevant to a discrimination task. Observers had to attend to all of the Gabors in the sequence and explicitly discriminate their color by means of a forced choice task (see Methods). If observers would combine orientation based on color, because the Gabors have to be discriminated by color, then serial dependence should only occur when the last stimulus within the trial sequence was of the same color as the one to be reported.

Sixteen observers performed the discrimination task with an overall accuracy of 92% and performed the orientation adjustment task with a standard deviation of errors of 9.47° ± 7.05° and an average adjustment time of 1.99 ± 0.99 seconds. As in Experiment 1, we considered serial dependence for *target* and *non-target* stimuli, as well as *within-* and *between-trial* effects. In the *within-trial* analysis, serial dependence was significant when the previous stimulus in the sequence had both the *target* color (α = 1.92°, *p* < 0.001) and the *non-target* color (α = 2.40°, *p* < 0.001), with no significant difference between the two (*p* > 0.05) (see Figure 2B). Similarly, in the *between-trial* analysis, the orientation of the stimulus reported on the previous trial induced systematic serial dependence, independently of whether the color of the stimulus reported on the previous and current trials was the same (*target* condition, α = 1.50°, *p* < 0.001) or different (*non-target* condition, α = 1.56°, *p* < 0.001; *target* vs. *non-target*: *p* > 0.05).

The results of Experiment 2 indicate that serial dependence for orientation is independent of changes in the stimulus color, even when observers are explicitly asked to discriminate two stimuli based on their color.

## Discussion

We investigated the role of a secondary feature (color) in serial dependence for orientation. Previous research has reported no influence of a secondary feature (Ceylan et al., 2021; Kim et al., 2020), but, in these cases, the secondary feature was entirely irrelevant to the task. In contrast, we made the color feature relevant to the secondary task. In Experiment 1, observers performed a color-detection task where color also indicated the color of the relevant stimuli for the adjustment task. In Experiment 2, observers had to discriminate the color of each stimulus, but the color was not informative for the orientation adjustment task.

We hypothesized that, by explicitly asking observers to perform a task based on color, serial dependence in orientation would be affected by color, indicating that the two features were integrated, as in object-level representations. This would be even more likely to occur when observers had to discriminate the stimuli based on color. However, our two experiments confirmed previous findings showing that serial dependence in orientation is independent of changes in the stimulus color.

In Experiment 1, serial dependence was affected by the target color, but this is likely because the target color also indicated the orientation to attend during the trial sequence. Hence, stimuli that were attended to in the past were of the same color as the one tested for serial dependence. This is in line with many studies showing a clear role of attention in serial dependence, with the typical observation that previous stimuli induce a bias when they are attended to (Fischer & Whitney, 2014; Fritsche & de Lange, 2019; Pascucci et al., 2019). As mentioned, in Experiment 1, we did not test serial dependence for stimuli of the unattended color. This leaves open the possibility that within-trial serial dependence could occur separately for stimuli of both the target color and the non-target color.

The results of Experiment 2 then demonstrated that, when stimuli of both colors are relevant, serial dependence occurs independently of the stimulus color. That is, even though observers were asked to discriminate the stimulus color, the orientation of the previous stimulus within the trial caused a bias on the current response, completely independent of whether the color was the same or not. Adding to the results of Experiment 1, this finding supports the conclusion that serial dependence in orientation is largely unaffected by changes in other features.

In both experiments, we replicated serial dependence effects from stimuli reproduced far back in time and followed by an intervening sequence of new stimuli that are not reported (Pascucci et al., 2019; Pascucci & Plomp, 2021). There are two important observations to make regarding this finding. First, this and similar results may appear surprising because serial dependence is believed to be a function of time, and the last reported stimulus was far back in time, followed by several other intervening stimuli, and also considering to the typically large *n* – 1 effects reported in many studies. We believe that the sequential no-report paradigm used here and in previous works may isolate two different forms of serial dependence, one that is largely driven and strengthened by processes related to behavioral reports (e.g., memory, decision-making) and the other that is induced by the stimulus even in the absence of a report. These two forms of serial dependence may operate at different time scales and be differentially affected by the time interval and the number of intervening stimuli between the inducer and the test stimulus. The second observation is that, although previous work has typically reported repulsive biases within trials and attractive ones between trials (Pascucci et al., 2019; Pascucci & Plomp, 2021), we found attractive biases (or no effect) for both (see also Abreo, Gergen, Gupta, & Samaha, 2023). Although the nature of repulsive biases in these tasks is still a matter of debate (Pascucci et al., 2023), one potential explanation is that what determines whether repulsion or attraction is seen is the task relevance of the stimulus or the role it plays in the task (e.g., Rafiei, Chetverikov, Hansmann-Roth, & Kristjánsson, 2021; Rafiei, Hansmann-Roth, et al., 2021). In prior work where the stimuli in the trial sequence were not relevant for any additional task, observers may have expected the relevant stimulus later in the sequence and paid less attention to the rest, thus promoting repulsive effects. In the current work, the additional task may have increased the relevance and level of attention during the entire sequence. Although this remains a possibility, further work is needed to clarify the nature of repulsive and attractive biases in these particular paradigms.

The main aims and the manipulation type here are reminiscent of the literature on feature binding and integration. To perceive the continuity of objects, the brain must conjointly represent and bind together multiple visual features that lie close in space and time, combining multiple features to represent coherent objects (Treisman, 1996). There are several views on this topic. One view proposes that the integration of visual features is initiated automatically, driven by the mere co-occurrence of stimulus features, but is then strongly mediated by task context and attention (Hommel, 2004; see also Ho, Abel, Correa, Littman, Cohen, & Griffiths, 2022). In the “relevance filter” model (Bundesen, 1990; Norman, 1968), for example, individual features are weighted and integrated into persisting “object files” depending on their task relevance and the current attentional set (Hommel & Colzato, 2004). Although the relation between serial dependence and object processing is still not well understood (Pascucci et al., 2023), our results can be tentatively contextualized within this framework, suggesting that feature integration (e.g., of color and orientation) is largely mediated by task context (Fischer et al., 2020; Hommel, 2004) and is not revealed by simply adding a secondary feature in serial dependence tasks.

Recent work has, however, shown that in other conditions serial dependence can be influenced by contextual features and object-level information (Collins, 2021; Fischer et al., 2020). For example, Fischer and colleagues (2020) used a paradigm in which two clouds of moving dots with different colors were memorized at the same time. A post-cue indicated the color of the relevant clouds on each trial. In this paradigm, serial dependence in motion direction judgments occurred only when the post-cued color was the same on the previous and present trials. This suggests that, when feature binding is necessary to select between competing representations in working memory, serial dependence occurs at the level of integrated features. Similarly, when visual judgments involve complex aspects of objects, such as emotional expressions, that can only be extrapolated through the combination of elementary features, serial dependence occurs at the object level and for conjunctions of features (Collins, 2021). Hence, even if we explicitly asked observers to distinguish the stimuli based on a secondary feature, our paradigms may not have fulfilled the necessary conditions to observe serial dependence at the level of objects and integrated features.

We propose that our findings reflect an observer's tendency to use minimal but sufficiently detailed representations to perform a perceptual task—that is, the information that propagates from one trial to the next depends on the representation required by the task (Ceylan et al., 2021; Kwak & Curtis, 2022). Features such as orientation can be reduced to a compact and low-dimensional format that allows for the sparing of unnecessary perceptual and memory resources (e.g., the orientation of a stimulus can be represented as a tilted line, discarding other irrelevant features) (Kwak & Curtis, 2022). As a consequence, serial dependence in these and similar paradigms may operate mostly at the level of low-dimensional abstract “codes” that are independent of the object and the other features it contains (Ceylan et al., 2021). Although this might be beneficial in terms of resource optimization in everyday vision, where objects and features are truly continuous and temporally correlated, it prevents feature integration and leads to object-independent serial dependence when the relationship between objects and features is experimentally altered.
